# Exhaustion of NK cells and interferon activation in anti-MDA5^+^ dermatomyositis are associated and determine the development of ILD

**DOI:** 10.3389/fimmu.2025.1697803

**Published:** 2025-12-08

**Authors:** Chenyi Shao, Yan Zhen, Nana Xia, Xueliang Zhang, Ninghui Yan, Mengmeng Zhou, Yiyuan Guo, Limin Tan, Qiang Guo

**Affiliations:** 1 Department of Rheumatology, Shanghai Jiao Tong University School of Medicine Affiliated Renji Hospital, Shanghai, China; 2 Vanke Bilingual High School, Shanghai, China; 3 Department of Rheumatology, Jiading District Central Hospital Affiliated Shanghai University of Medicine & Health Sciences, Shanghai, China

**Keywords:** dermatomyositis (DM), interstitial lung disease (ILD), natural killer (NK) cell, interferon, single-cell sequencing, apoptosis

## Abstract

**Objectives:**

This study aimed to investigate the role of natural killer (NK) cell and interferon (IFN) signaling in the peripheral circulation of anti-MDA5^+^ dermatomyositis (DM) patients, particularly in the context of interstitial lung disease (ILD) development.

**Methods:**

Peripheral blood mononuclear cells (PBMCs) from six newly diagnosed, active anti-MDA5^+^ DM patients (3 with ILD, 3 without ILD) were analyzed using single-cell RNA sequencing (scRNA-seq), focusing on NK cell features. The correlation between serum cytokine levels and ILD severity assessed by HRCT scores was further analyzed. Flow cytometry in an independent cohort validated NK cell apoptosis, and *in vitro* experiments evaluated IFN-induced apoptosis in NK-92 cells.

**Results:**

ScRNA-seq revealed widespread activation of IFN responses in PBMCs from anti-MDA5^+^ DM-ILD patients, with pronounced upregulation in innate immune cells (e.g., NK cells and monocytes). A reduction in circulating NK cell proportions was observed in ILD patients. Serum levels of cytokines (IL-1β, IL-2, IL-4, IL-5, IL-10, IL-12P70, TNF-α, and IFN-α) positively correlated with the radiologic severity of ILD as quantified by HRCT scores. Flow cytometry confirmed significantly decreased NK cell counts and increased apoptosis in the ILD group. PBMCs from healthy donors exposed to an ILD-like cytokine milieu exhibited upregulated IFN pathway genes (ISG15, MX2, IFIT3), EIF2AK2 (encoding protein kinase R), and apoptosis-related genes (BCL2, BAX2). *In vitro*, IFN stimulation directly induced apoptosis in NK-92 cells.

**Conclusion:**

Excessive IFN activation drives NK cell apoptosis in anti-MDA5^+^ DM-ILD, contributing to NK cell depletion and ILD development. IFN-related biomarkers may serve as valuable indicators for assessing ILD severity in these patients.

## Introduction

Idiopathic inflammatory myopathies (IIMs), are a group of autoimmune systemic diseases characterized by diverse clinical manifestations affecting the skin, muscles, joints, and lungs (1). Among these, the lungs are one of the most frequently involved extramuscular organs, with interstitial lung disease (ILD) reported in 20-86% of patients (2). ILD is associated with poor prognosis and increased mortality (3, 4), particularly in rapidly progressive ILD (RP-ILD), where respiratory function deteriorates within 2–3 months (5, 6). Anti-MDA5^+^ dermatomyositis (anti-MDA5^+^ DM), a distinct subtype of DM, is characterized by serum reactivity against the autoantigen MDA5 and unique cutaneous manifestations such as Gottron’s papules and heliotrope rash, often with absent or atypical myositis symptoms (1, 7, 8). Notably, 90-95% of anti-MDA5^+^ DM patients develop ILD, with a high propensity for RP-ILD, making it a major clinical challenge due to its rapid progression and high mortality (1).

MDA5, a pattern recognition receptor (PRR), plays a critical role in antiviral immunity by recognizing viral RNA and triggering interferon (IFN) production (9, 10). In patients with anti-MDA5^+^ DM, aberrant activation of MDA5 triggers the overproduction of interferons, particularly type I interferons (6, 11), while anti-MDA5 autoantibodies may form immune complexes (ICs) that further amplify IFN signaling through neutrophil extracellular trap (NET) release (12, 13). Elevated levels of type II IFN (IFN-γ) have also been observed in anti-MDA5^+^ DM patients with RP-ILD, suggesting a role for dysregulated IFN signaling in disease pathogenesis (14, 15). Therefore, continuous activation of the interferon signaling pathway may drive immune-mediated lung tissue damage, contributing to ILD development (12). Additionally, MDA5 activation promotes pro-inflammatory cytokine and chemokine release, exacerbating lung inflammation and fibrosis (12, 16, 17).

Despite the established role of dysregulated IFN signaling in the pathogenesis of anti-MDA5+ DM-ILD, the specific contribution of innate immune cell subsets, particularly natural killer (NK) cells, to the development and progression of lung injury remains poorly defined. Previous studies have highlighted the critical role of NK cell dysregulation in this process. In a cohort of amyopathic dermatomyositis-associated ILD patients, our group identified a distinct immunological profile characterized by activated CD45RA^+^HLA-DR^+^CD8^+^ T cells and reduced CD56^dim^ NK cell proportions, which correlated with rapid ILD progression and increased mortality (18). Further supporting this, our recent work demonstrated that the depletion of NK cells is of great value in predicting the progression of pulmonary fibrosis in patients anti-MDA5^+^ DM-ILD (19). In anti-MDA5^+^ DM patients, the significantly reduced levels and an inhibitory phenotype of peripheral NK cells are correlated with heightened disease activity and adverse prognosis (20). These findings suggest that NK cell dysfunction or depletion may contribute to ILD pathogenesis.

To further elucidate the role of NK cells and its link to IFN signaling in anti-MDA5^+^ DM-ILD pathogenesis, we performed single-cell RNA sequencing (scRNA-seq) analysis on peripheral blood mononuclear cells (PBMCs) from patients with anti-MDA5^+^DM with and without ILD. Our analysis revealed widespread IFN pathway activation, particularly in innate immune cells such as NK cells and monocytes, alongside a reduction in circulating NK cell proportions and enhanced apoptotic signaling. These findings collectively implicate that IFN-induced NK cell apoptosis may be a critical mechanism driving NK cell depletion and ILD progression in anti-MDA5^+^ DM.

## Materials and methods

### Ethical statement

This study was conducted in accordance with the Declaration of Helsinki and approved by the Research Ethics Committee of Renji Hospital (ID:2013-126). All participants had been informed of the purpose of the study and provided written informed consent for study enrolment and blood collection.

### Patient selection and data collection

For this study, peripheral blood samples were collected from anti-MDA5^+^ DM patients who fulfilled the EULAR/ACR classification criteria of IIM at the Department of Rheumatology of the Renji Hospital of Shanghai Jiao Tong University (21). The semiquantitative detection of anti-MDA5 antibody was performed using EUROLINE Autoimmune Inflammatory Myopathies 16 Ag (IgG) (Euroimmun, Germany). HRCT confirmed ILD status (presence/absence) based on radiological criteria at enrollment (22, 23), with no progression to ILD observed in the non-ILD group during follow-up monitoring. HRCT scans were performed on a GE Optima CT680 scanner during full inspiration, utilizing a volumetric acquisition protocol with 1.25 mm slice reconstruction. Patients with active infections, malignancies, or other lung diseases were excluded. Clinical data and blood samples were collected prior to immunosuppressive therapy for patients who provided PBMC samples for sequencing analysis. Furthermore, during the clinical follow-up, no evidence of CMV reactivation or active infection was detected through routine clinical monitoring.

Demographic details for the independent validation cohorts can be seen in **Supplementary Table 1**. For the sample validation cohort 1, HRCT scores were additionally collected for the assessment of ILD severity (24, 25). All scans were independently evaluated by two thoracic radiologists, each with over ten years of experience, who were blinded to the clinical and laboratory data. The radiologists assessed the presence, extent, and distribution of CT findings–including ground-glass attenuation (GGA), consolidation, traction bronchiectasis or bronchiolectasis, and honeycombing–in accordance with the Fleischner Society criteria (26). A composite score was derived for each patient (see **Supplementary Data S1** for detailed scoring methodology), integrating both the severity of the fibrotic pattern and its spatial extent to provide a comprehensive quantification of ILD severity.

### Single-cell RNA sequencing and data processing

Cryopreserved PBMCs were processed using the 10x Genomics Chromium platform. Sequencing libraries were constructed and analyzed via Cell Ranger (GRCh38-1.2.0 reference) with quality filtering.

Integrated scRNA-seq data (Seurat) underwent normalization, dimensionality reduction (UMAP), and cluster identification. Differential gene expression (Seurat: log2FC>0.25, adj.p<0.05), GO and KEGG pathway enrichment (clusterProfiler), and gene set (GSVA/GSEA) were analyzed. PPI networks (Cytoscape) identified hub genes. Gene set enrichment analysis was further complemented using the UCell package (27) to compute single-sample gene set enrichment scores based on gene expression rankings via the Mann-Whitney U statistic. More detailed methods can be found in **Supplementary Data S1**.

### *In vitro* experiments

1*10^7^ PBMCs from healthy volunteers were cultured in six-well plates and then treated with RPMI 1640 culture medium containing 20% plasma of patients with anti-MDA5^+^ DM (ILD/non-ILD) for 24h.

NK-92 cells were maintained in the special culture medium containing β-MEM, 10% fetal bovine serum, 10% horse serum, 0.2 mmol inositol, 0.1 mmol β-mercaptoethanol and 10 ng/ml IL-2 in 12-well plates, with 5*10^5^ cells per well. The cells were cultured for 0, 24, 48 and 72 hours with different concentrations of IFN-α (PeproTech, USA; 0, 50 and 100 ng/ml) and then assessed by flow cytometry. For plasma stimulation assays, NK-92 cells were cultured under the same conditions and exposed to 20% plasma pooled from healthy donors, anti-MDA5+ DM patients with ILD, or anti-MDA5+ DM patients without ILD. After 72 hours of incubation, cells were harvested and subjected to flow cytometry for evaluation of surface markers and functional molecules.

### Real-time fluorescent quantitative PCR

For the plasma incubation experiments, total RNA was extracted from healthy donor-derived PBMCs following incubation using the Trizol reagent method. RNA purity was confirmed by an OD260/280 ratio of 1.8-2.0. Following the manufacturer’s protocol (Serumwerk Bernburg AG), reverse transcription was performed at 37 °C for 15 minutes followed by 85 °C for 5 seconds. Real-time fluorescent quantitative PCR cycling conditions included an initial denaturation at 95 °C for 30 seconds, followed by 40 cycles of 95 °C for 5 seconds and 60 °C for 30 seconds. Gene expression levels were quantified using the 2-ΔΔCT method, calculating the relative expression of target genes.

### Detection of serum cytokines

Serum cytokine concentrations (IL-1β, IL-2, IL-4, IL-5, IL-6, IL-8, IL-10, IL-12p70, IL-17A, TNF-α, IFN-α, and IFN-γ) were quantified using cytometric bead array (CBA, Cellgene Biotech). Serum was isolated from whole blood, incubated with cytokine capture beads, and analyzed on a BD FACSCanto II flow cytometer. Data were processed using FCAP Array v3.0 (BD Biosciences). All cytokine assays were conducted within the clinical laboratory of the hospital, adhering to standardized operational protocols and ensuring the reproducibility of the results.

### Flow cytometry

Fresh blood samples were processed within 2 hours for PBMC isolation. Cells were incubated with an Fc receptor blocker (4 °C, 10 min), stained with fluorescent antibodies (4 °C, 20 min), and analyzed on a BD FACSCanto II. Lymphocyte subsets were identified as follows: CD45^+^ (lymphocytes), and CD56^+^CD3^−^ (NK cells). Apoptosis was assessed using Annexin V (live: Annexin V^−^7-AAD^−^; early apoptotic: Annexin V^+^ 7-AAD^−^). Data were analyzed using FlowJo v10.8.1.

### Statistical analysis

Data were analyzed using R (v4.3.0), SPSS (v27.0), and GraphPad Prism (v9.5). The presentation of data was based on their distribution: continuous variables are reported as mean with standard deviation or median with interquartile range, and categorical variables as counts and percentages. For group comparisons, the Student’s t-test or Mann-Whitney U test was applied to continuous data, while the Chi-square test or Fisher’s exact test was used for categorical variables, as appropriate. Pearson correlation assessed relationships between continuous variables. Significance was set at p<0.05.

## Results

### Cohort characteristics

To investigate the immune cell dynamics associated with ILD in anti-MDA5^+^ DM, we collected PBMCs from six newly diagnosed, treatment-naive anti-MDA5^+^ DM patients (3 with ILD, 3 without ILD). ILD diagnosis was confirmed by HRCT, and clinical characteristics were summarized in **Table 1**.

**Table 1:** Clinical and laboratory data for groups.

Group	ILD group			Non-ILD group		
Sample ID	ILD_1	ILD_2	ILD_3	NonILD_1	NonILD_2	NonILD_3
Age (years)	51	49	56	44	64	62
gentle	male	female	female	female	female	female
Clinical symptoms	Cough and wheeze; Gottron’s sign; Heliotrope rash; Periungual erythema; Oral ulcers	Rash on the hands, forehead, back, extensor surfaces of both elbows, and lateral thighs; Cough with expectoration; Joint swelling and pain; Muscle pain in the limbs	Purple-red rash on the face; Cough; V-shaped rash on the anterior neck	Cutaneous vasculitis; Occasional joint swelling and pain	Purple-red rash on bilateral fingers with joint pain; Facial erythema; Gottron’s sign; Periungual keratosis	Rash on the extensor surfaces of both hands and elbows, periorbital rash; Finger ulcers; Fatigue; cough and wheeze
Antibody	anti-MDA5^+^	RO52^+^ anti-MDA5^+^ ANA 1:80	RO52^+++^ anti-MDA5^+++^	anti-MDA5^+^	RO52^+++^ anti-MDA5^+++^	anti-MDA5^+^
Imaging	Increased pulmonary markings with multiple patchy and linear opacities in the lungs, more prominent in the left lung, indicative of bilateral pulmonary inflammation.	Bilateral pulmonary effusion with multiple band-like opacities in the right middle lobe, lingula of the left upper lobe, and both lower lobes.	Subpleural scattered multiple band-like and patchy consolidations in both lungs, more severe in the lower lobes, with scattered spots and nodules. Bilateral interstitial pneumonia.	Clear lung fields with no evident interstitial changes in both lungs.	Clear lung fields with no evident interstitial changes in both lungs.	Clear lung fields with a small amount of fluffy opacities in the left lower lobe.
Others	Skin biopsy indicates inflammatory cell infiltration.					

### Single-cell profiling reveals NK cell depletion in ILD patients

**Figure 1:**
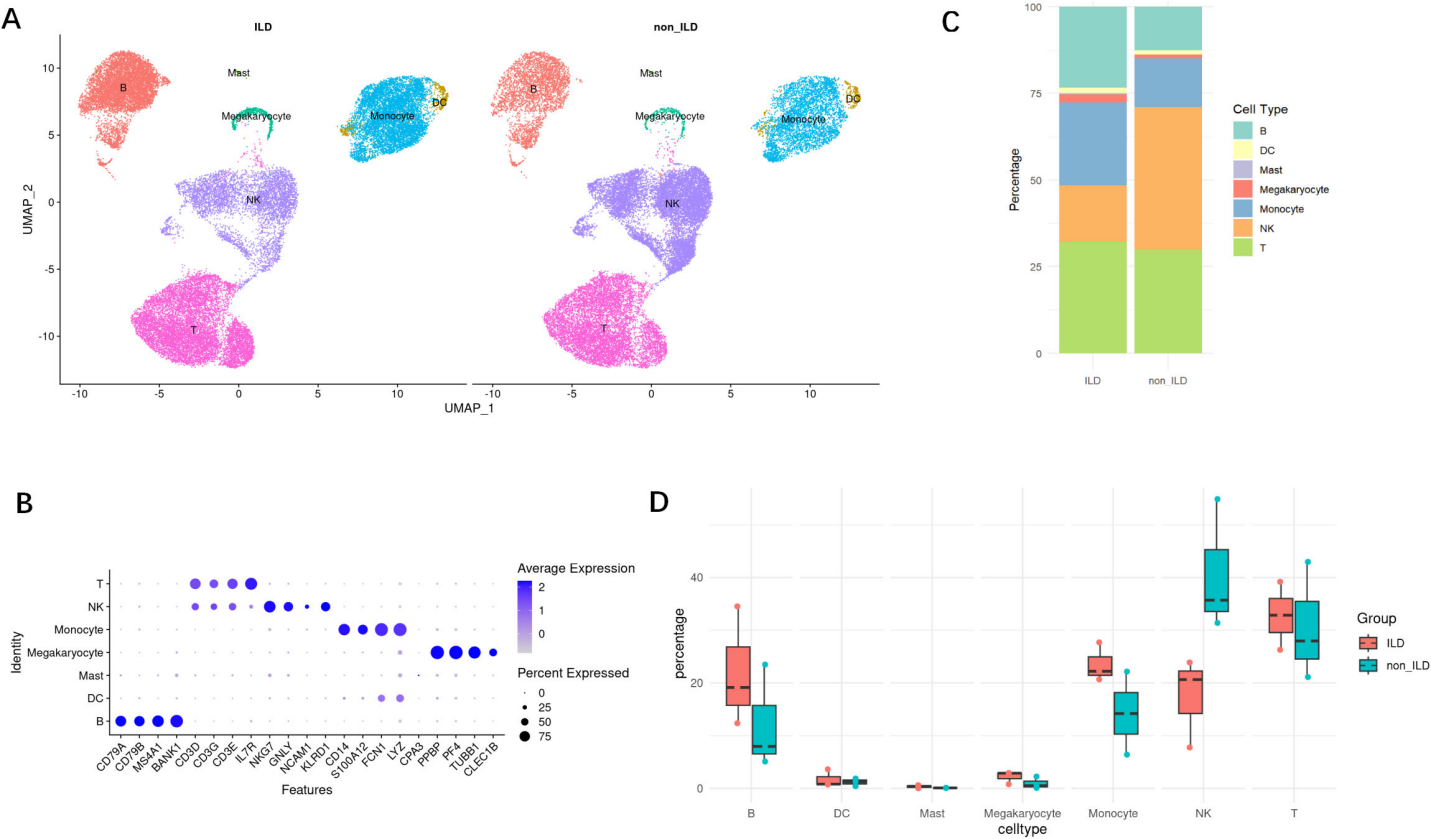
Single-cell atlas of PBMCs from anti-MDA5^+^ DM with or without ILD. **(A)** UMAP plot of main cell types in ILD and non-ILD groups. **(B)** Dot plot of cell group-specific marker genes. **(C)** Stacked bar charts of the proportions of major cell types in ILD and non-ILD groups. **(D)** Box plots of the proportions of cell subtypes in ILD and non-ILD groups.

Following data preprocessing and quality control, we obtained a total of 59,427 immune cells from PBMCs derived from six subjects, comprising 30,978 cells from the ILD group and 28,449 from the non-ILD group. Using unsupervised graph-based clustering analysis and marker recognition, we identified seven major cell types across all PBMC samples, including B cells, T cells, NK cells, monocytes, dendritic cells, mast cells, and megakaryocytes (**Figures 1A, B**). Comparative analysis revealed a relative depletion of NK cells in the ILD group compared to the non-ILD group (16.2% vs. 41.2%). Concurrently, we observed an enrichment of B cells (23.3% vs. 12.6%) and monocytes (24.0% vs. 14.2%) in the ILD group (**Figures 1C, D**).

### The ILD group exhibits a pronounced IFN signature

Through inter-group differential analysis of all cells between the ILD and non-ILD groups, we identified 2,359 differentially expressed genes (DEGs) in the ILD group, with 907 genes upregulated and 1,452 genes downregulated (**Supplementary Figure S1A**). Gene Ontology (GO) enrichment analysis (**Supplementary Figure S1B**) of these DEGs revealed that upregulated DEGs were predominantly involved in viral response, activation of the immune system, signal transduction, and production of cytokines related to inflammation. Conversely, downregulated DEGs were significantly enriched in processes related to T cell activation, differentiation, and T cell receptor signaling pathways.

**Figure 2:**
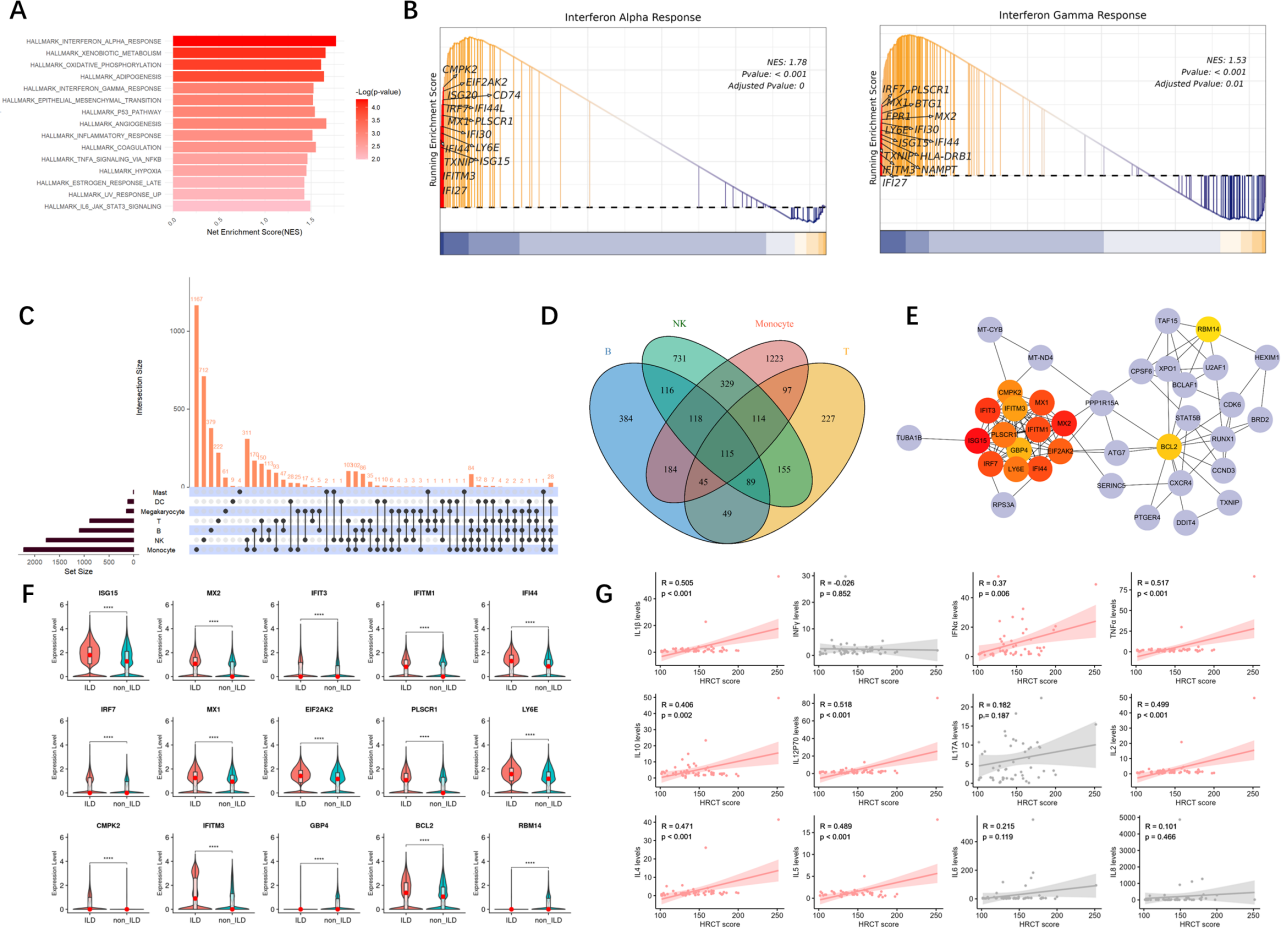
The ILD group exhibits a pronounced IFN signature. **(A)** The bar graph of the differential gene sets between the ILD group and the non-ILD group, sorted by significance. **(B)** GSEA of IFN-α and IFN-γ signaling pathways. The plots show the enrichment profiles with the top 15 leading-edge genes (key contributors to the enrichment signal) highlighted. NES, normalized enrichment score; FDR, false discovery rate. **(C)** UpSetR plot of intersecting genes. ****: p<0.0001. **(D)** Venn diagram of common DEGs in four major types of lymphocytes. **(E)** Protein-Protein Interaction analysis of core genes, with darker colors indicating higher MCC (Maximal Clique Centrality) scores. **(F)** Violin plots with embedded boxplots display the expression profiles of representative DEGs across groups. Red dots indicate median values. Group comparisons were performed using the Mann-Whitney U test. **(G)** Correlation analysis between serum cytokine levels and HRCT scores. Each point represents an individual patient. (R represents the correlation coefficient; p indicates the significance level.).

Subsequent Gene Set Enrichment Analysis (GSEA) (**Figure 2A**) revealed significant enrichment of several key biological pathways and cellular processes in the ILD group, including interferon-alpha (IFN-α) and interferon-gamma (IFN-γ) response, oxidative phosphorylation, apoptosis, and tumor necrosis factor-alpha (TNF-α) signaling via NF-κB. Notably, the IFN-α response pathway (**Figure 2**) was significantly upregulated in the ILD group (NES = 1.77, adjusted p-value <0.001), with key genes such as IFI27, IFITM3, and TXNIP highlighted in the analysis. The IFN-γ pathway was also found to be significantly upregulated in the ILD group.

Through DEG analysis across various cell subpopulations between the ILD group and the non-ILD group, we identified a considerable number of differentially expressed genes (**Supplementary Figure S2A**). Among these, monocytes demonstrated the most substantial number of DEGs, totaling 2,225, with 751 upregulated and 1,474 downregulated genes, followed by NK cells, which exhibited 1,767 DEGs, comprising 956 upregulated and 811 downregulated genes. B cells had 1,100 DEGs, with 170 upregulated and 930 downregulated. T cells showed 891 differentially expressed genes, with 398 upregulated and 493 downregulated. Enrichment analysis of DEGs across these cell subgroups reflected functional differences in cellular behavior between the ILD and non-ILD phenotypes of anti-MDA5^+^ DM (**Figure 2B**). We identified 115 common DEGs across the four main lymphocyte subgroups: B cells, T cells, monocytes, and NK cells (**Figures 2C, D**). Protein-Protein Interaction (PPI) analysis yielded 15 core genes (**Figure 2E**), most of which are associated with the interferon pathway, including ISG15, MX1, MX2, IFIT3, IFITM1, IFITM3, IRF7, LY6E, and CMPK2. Expression levels of these genes were significantly higher in the ILD group compared to the non-ILD group (**Figure 2F**).

Additionally, we collected an independent cohort of PBMCs samples to explore the correlation between serum cytokine levels and HRCT scores, and revealed a positive correlation with multiple cytokines, including TNF-α, IFN-α, IL-1β, IL-2, IL-4, IL-5, IL-10, and IL-12P70 (p < 0.05). This suggests that these cytokines play crucial roles in the pathogenesis of ILD (**Figure 2G**).

### IFN responses are enriched in innate immune cells

Using the UCell gene set scoring method, we further explored the activation of the interferon pathway across various cell subpopulations to identify the principal cellular contributors to IFN signaling.

**Figure 3:**
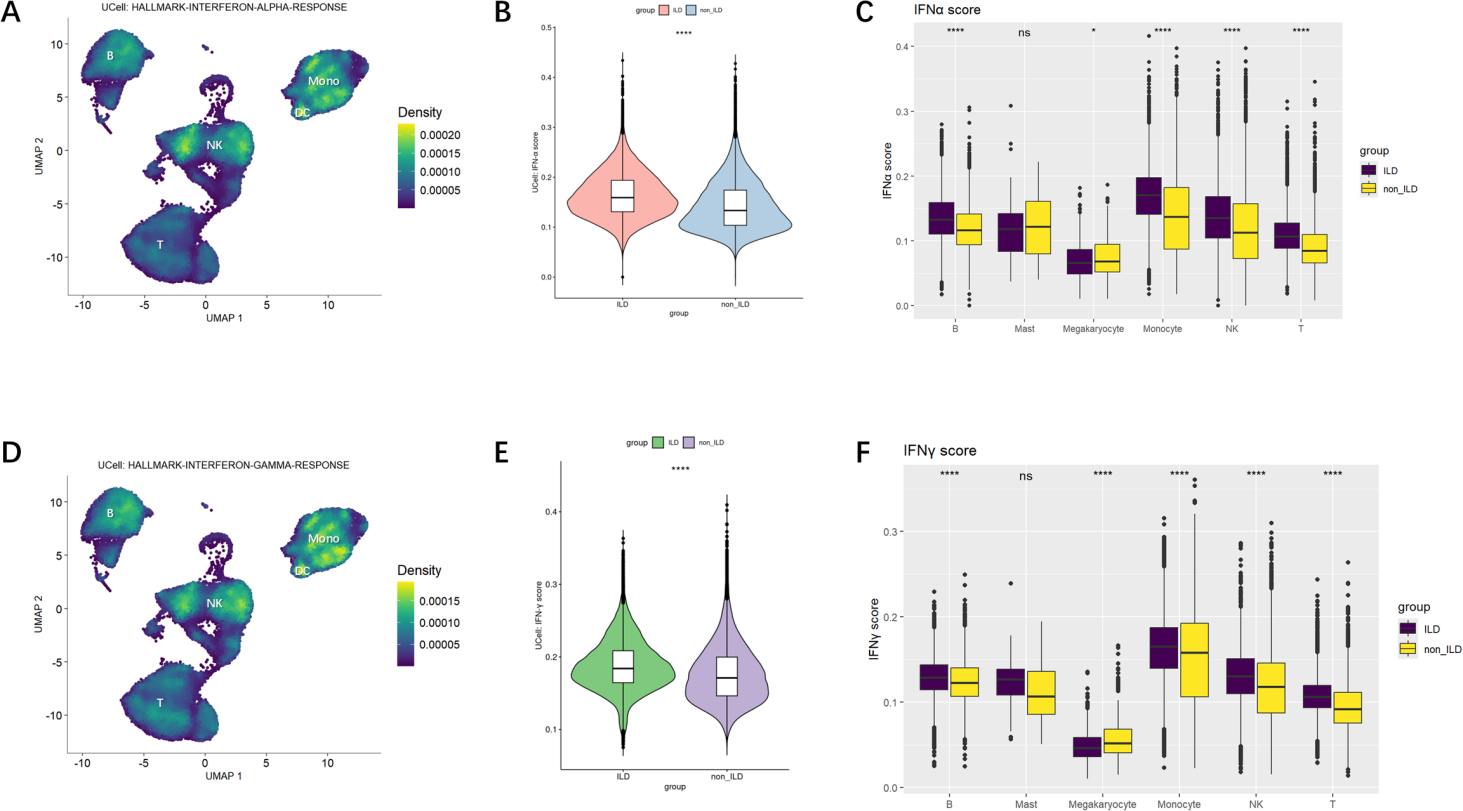
IFN-α and IFN-γ Gene Set Scoring. **(A)** Ucell calculation of enrichment scores for IFN-α. The yellower the color, the higher the density score, indicating a higher enrichment score. **(B)** Violin plot of comparison of IFN-α gene set scores across groups. **(C)** Boxplot of differences in IFN-α scores among cell subpopulations. **(D)** Ucell calculation of enrichment scores for IFN-γ. **(E)** Violin plot of comparison of IFN-γ gene set scores across groups. **(F)** Boxplot of differences in IFN-γ scores among cell subpopulations. Statistical comparisons were performed using the Mann-Whitney U test. *p<0.05; ****p<0.0001.

We discovered that gene sets regulated by IFN-α were predominantly enriched in NK cells and monocytes, with partial enrichment in B cells (**Figure 3A**). Comparative analysis between groups (**Figures 3B, C**) revealed that the IFN-α gene set scores were significantly higher in the ILD group of anti-MDA5^+^DM patients compared to the non-ILD group. Subsequent evaluation of IFN-α scores across individual cell subpopulations showed that innate immune cells, particularly monocytes and NK cells, generally exhibited higher IFN-α scores. Although B cells and T cells had relatively lower IFN-α scores, they also demonstrated statistically significant differences between groups. Megakaryocytes showed lower IFN-α scores in the ILD group compared to the non-ILD group; however, the differences in IFN-α scores between the two groups were not statistically significant in mast cells.

Similar patterns were observed when analyzing the expression of the IFN-γ gene set across various cell types (**Figure 3D**), which were also mainly enriched in NK cells and monocytes. **Figures 3E, F** indicated that IFN-γ gene set scores were significantly higher in the ILD group compared to the non-ILD group. When comparing IFN-γ gene set scores among subpopulations, IFN-γ signaling was elevated in monocytes and NK cells.

To further explore the relationship between inflammation and disease pathogenesis, we concurrently analyzed the enrichment scores of inflammasome-related gene sets. As shown in **Supplementary Figure S3A**, the NLRP3 inflammasome pathway was predominantly enriched in monocytes. At the individual cell level (**Supplementary Figures S3B, C**), we observed activation of the inflammasome pathway across multiple cell subpopulations in the ILD group compared to the non-ILD group, suggesting a broad inflammatory activation state in patients with pulmonary involvement.

### The reduced NK cells and the increased apoptosis signal in the ILD group are closely related

**Figure 4:**
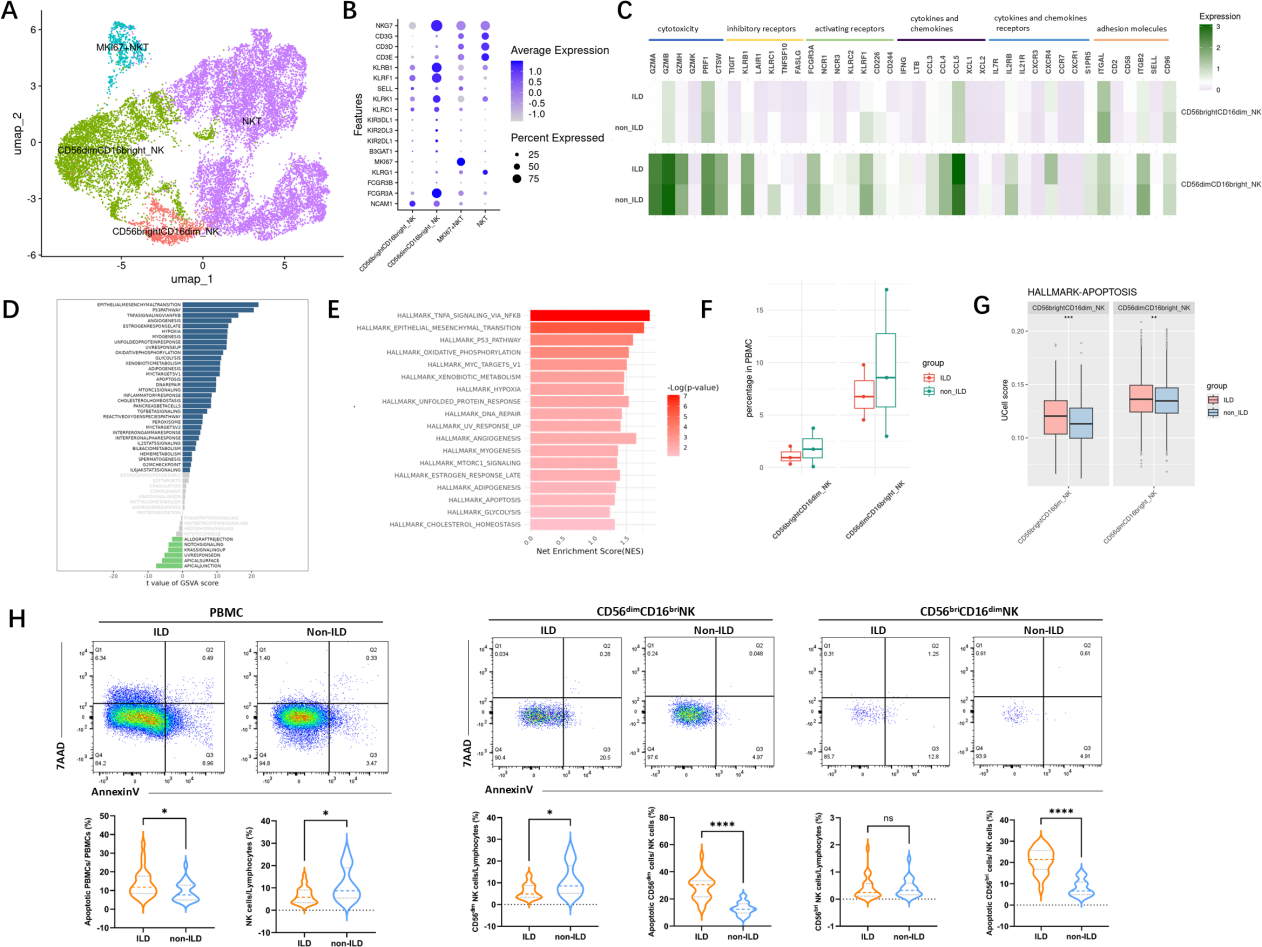
Characterization of NK cells in the ILD and non-ILD groups. **(A)** UMAP visualization of NK cell subsets. **(B)** Dot plot showing expression of subset-specific marker genes in NK cell subpopulations. **(C)** Classical markers of NK subsets including activation, inhibition, cytotoxicity, and cytokine. **(D)** GSVA barplot of the ILD and non-ILD groups. **(E)** GSEA barplot of the ILD and non-ILD groups. **(F)** Box plots of the proportions of NK cell subsets in ILD and non-ILD groups **(G)** Ucell score of apoptotic gene sets in different NK cell subsets and groups. **(H)** Ucell score of apoptotic gene sets in different cell subsets and groups. **(H)** Comparison of NK cell subsets proportions and the proportion of apoptotic cells between the ILD and non-ILD groups (see **Supplementary Figure S4** for gating strategy schematic). Statistical analysis was performed using the Mann-Whitney U test. *: p<0.05; **: p<0.01; ***: p<0.001.****: p<0.0001; ns: not significant.

To further investigate the heterogeneity of NK cells, we performed subset re-clustering and UMAP dimensionality reduction on NK cells (**Figure 4A**). Human peripheral blood NK cells were classified into two developmentally related but functionally distinct canonical subsets based on the expression of classical NK cell marker genes: CD56^dim^CD16^bright^ NK cells and CD56^bright^CD16^dim^ NK cells (**Figure 4B**). A cluster expressing high levels of CD3D was identified as NKT cells and excluded from subsequent subset analyses. Comparative analysis based on classical NK cell markers–including those related to activation, inhibition, cytotoxicity, and cytokines–was conducted (**Figure 4C**). CD56^dim^CD16^bright^ NK cells, the predominant subset in human peripheral blood, exhibited relatively higher expression levels of genes associated with cytotoxicity, inhibitory receptors, as well as chemokines and adhesion molecules.

Using GSVA and GSEA analysis (**Figures 4D, E**), we compared the differences in gene expression and signaling pathways in NK cells between ILD and non-ILD patients. We observed significant upregulation of the epithelial-mesenchymal transition, P53 pathway, TNF-alpha signaling, interferon-alpha response, interferon-gamma response, and apoptosis signaling pathways in the NK cell population of the ILD group. Our previous cell proportion analysis indicated a trend toward a reduced proportion of NK cells in the ILD group. Although the limited sample size in our single-cell RNA sequencing data precluded statistical significance, further analysis of NK subsets revealed elevated apoptotic signaling in both NK cell subsets in the ILD group (**Figures 4F, G**).

We further validated this observation in an independent cohort. As shown in **Figure 4H**, among patients with anti-MDA5^+^ DM, those with ILD exhibit a more pronounced depletion of NK cells compared to those without ILD, primarily characterized by a reduction in CD56dim NK cells. Furthermore, there was a significantly higher proportion of apoptotic NK cells in the ILD group.

### IFN induces NK cell apoptosis *in vitro*

**Figure 5:**
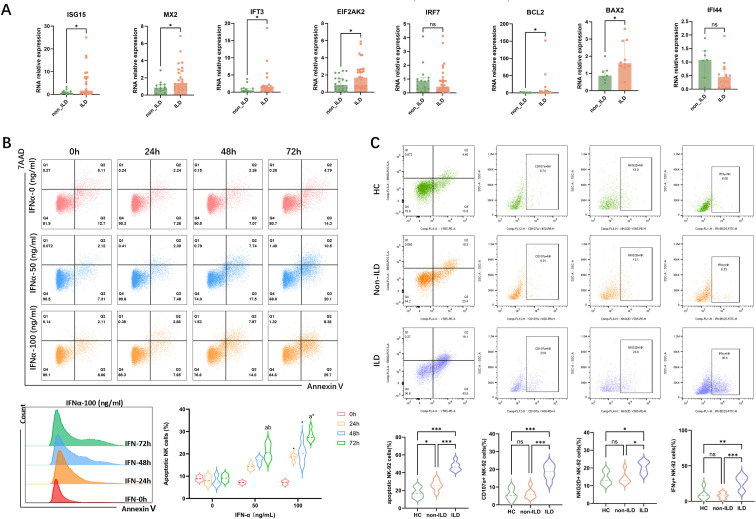
Impact of Interferon on Gene Expression in PBMCs and Apoptosis in NK-92 Cells. **(A)** The impact of plasma from patients with anti-MDA5^+^ DM with ILD/non-ILD on gene expression in PBMCs from healthy donors. Data are presented as median with IQR. Statistical analysis was performed using the Mann-Whitney U test. **(B)** IFN-α induces apoptosis in NK-92 cells in a dose- and time-dependent manner. For each concentration and time of interferon stimulation, six independent samples were repeatedly used to calculate the apoptosis expression of NK cells in different groups (n=6), and the results of each group were expressed by the median with IQR. Statistical analysis was performed using the Kruskal-Wallis test. (a: p<0.05 vs 0 (h); b: p<0.05 vs 24 (h)1; *: p<0.05 vs IFN-0 (ng/ml)). **(C)** Functional profiling of NK-92 cells following stimulation with plasma from healthy donors or anti-MDA5+ DM patients with or without ILD. Expression of NKG2D, CD107a, and IFN-γ was assessed. Six independent replicates (n=6) were used per group; data are shown as median with IQR, and the Kruskal-Wallis test was used for statistical analysis. *: p<0.05; **: p<0.01; ***: p<0.001.

Building on previous findings, we identified a significant correlation between cytokine levels in peripheral serum and ILD severity in anti-MDA5^+^ DM patients. Besides, patients with anti-MDA5^+^ DM-ILD exhibited a more pronounced depletion of NK cells with a higher proportion of apoptotic NK cells. To explore the impact of the disease cytokine milieu on PBMCs, plasma from anti-MDA5^+^ DM patients was categorized into ILD and non-ILD groups and used to stimulate PBMCs from healthy donors. Experimental results (**Figure 5A**) showed that PBMCs exposed to the ILD environment exhibited significantly increased expression of interferon pathway genes (ISG15, MX2, and IFIT3), EIF2AK2 (encoding protein kinase R), and apoptosis-related genes (BCL2, BAX2) compared to the non-ILD group.

To investigate the effect of increased interferon pathway activity on NK cell apoptosis, we conducted an *in vitro* stimulation experiment using the NK-92 cell line. Flow cytometry analysis (**Figure 5B**) after 72 hours of IFN-α stimulation revealed significant apoptosis in NK cells, particularly at a high concentration of 100 ng/ml. Importantly, the observed apoptosis was not due to general cytotoxicity, as cell viability remained unaffected across all IFN-α concentrations and time points tested (**Supplementary Figure S5B**). While some differences were not statistically significant due to sample size, the trend indicated that higher concentrations and longer stimulation periods generally increased NK cell apoptosis. It suggested that elevated interferon activity in patients with severe ILD may contribute to NK cell apoptosis through a specific mechanism rather than non-specific toxicity. We further evaluated the functional effects of plasma from healthy donors and patients with anti-MDA5+ DM, either with or without ILD, on NK-92 cells. As shown in **Figure 5C**, stimulation with plasma from ILD patients led to increased proportions of NK-92 cells expressing the activating receptor NKG2D, the degranulation marker CD107a, and the cytokine IFN-γ, suggesting enhanced NK cell activation and function under disease-relevant conditions.

## Discussion

Anti-MDA5^+^ DM is a clinically distinct subtype of idiopathic inflammatory myopathies (IIMs), characterized by a high prevalence of rapidly progressive interstitial lung disease (RP-ILD) and poor response to conventional immunosuppressive therapies (28). While overactivation of immune cells and dysregulated cytokine production are recognized as key pathological features, the precise mechanisms driving ILD in anti-MDA5^+^ DM remain elusive. Previous studies have predominantly focused on adaptive immune cells, such as T and B cells, leaving the role of innate immunity underexplored, particularly, the mechanisms of NK cells remain unclear.

Our study reveals that innate immune cells, particularly NK cells and monocytes, are the primary responders to interferon (IFN) signaling in anti-MDA5^+^ DM-ILD. Through single-cell RNA sequencing, we identified enriched IFN response and apoptotic pathways in NK cells from ILD patients. These findings were further supported by flow cytometry analysis, which demonstrated increased NK cell apoptosis and reduced circulating NK cell proportions in ILD patients. *In vitro* experiments demonstrated that IFN stimulation directly induces NK cell apoptosis, supporting the hypothesis that IFN-driven NK cell depletion plays a critical role in the development and progression of ILD in anti-MDA5^+^ DM patients.

While previous studies emphasized the role of adaptive immune cells (e.g., CD8^+^ T cells in Ye et al. (14)) or monocytes (e.g., He et al. and Shi et al. (29, 30)), our study highlights NK cell apoptosis as a previously underappreciated mechanism in ILD pathogenesis. To our knowledge, this is the first study to utilize single-cell sequencing to explore the association between IFN signaling and alterations in the immune cell landscape in the progression of ILD in anti-MDA5^+^ DM patients. It identifies the potential significance of enhanced IFN signaling and NK cell apoptosis in ILD, providing new insights into the disease mechanisms.

This study aligns with our recent findings that NK cell exhaustion can predict ILD progression in IIM patients, particularly those with anti-MDA5^+^ DM (19). Prior studies have also reported NK cell depletion in DM patients with severe pulmonary involvement (31). Meanwhile, a recent study also reported increased cell death signals in peripheral NK cells from DM patients compared to healthy controls (30). Our findings confirm significant NK cell depletion and increased apoptosis in the peripheral blood of anti-MDA5+ DM-ILD patients. This process appears to be driven by the prominent IFN signature in these patients, as evidenced by the positive correlation between serum IFN-α levels and ILD severity, and our *in vitro* model demonstrating IFN-induced NK cell apoptosis.

However, the observed reduction in circulating NK cells is likely multifactorial. In addition to apoptosis, cell migration may represent another mechanism, as evidenced by studies showing substantial NK cell infiltration in the lungs of patients with anti-synthetase syndrome (aSS) (32). Meanwhile, a recent study in anti-MDA5^+^ DM patients further indicated that monocytes and NK cells can be recruited to the lungs under the influence of chemokines, contributing to pulmonary inflammation (30). Therefore, we propose a collaborative model where the IFN-rich environment in anti-MDA5+ DM-ILD both primes NK cells for apoptosis and facilitates their trafficking to the pulmonary compartment. The migrated cells may subsequently contribute to local inflammation or undergo activation-induced cell death *in situ*, collectively leading to NK cell depletion in the periphery and potentially exacerbating lung injury. The net pathophysiological effect of NK cell loss is complex, as it may impair antiviral immune surveillance while potentially attenuating their pro-inflammatory tissue damage, with the balance likely determined by the local microenvironment. Our finding of increased NK cell apoptosis suggests that the IFN-rich milieu in anti-MDA5^+^ DM-ILD may actively shape the immune response by modulating NK cell availability.

The identification of IFN-induced NK cell apoptosis as a key mechanism in ILD pathogenesis has important therapeutic implications. IFN-α, IFN-β and IFN-γ induce the expression of transcription factors through activation of different JAK-STAT pathways, particularly classic interferon-stimulated genes (ISGs) (12). JAK inhibitors, such as tofacitinib (33), have emerged as promising therapeutic agents in this context. Prospective clinical trials reported that tofacitinib combined with glucocorticoids significantly improves survival rates and IFN scores in early anti-MDA5^+^ DM-ILD patients compared to conventional immunosuppressive treatments (33, 34). A clinical case study also found that tofacitinib treatment can reverse NK cell deficiency in dermatomyositis patients with rapidly progressive interstitial lung disease (35). Therefore, we propose that IFN signal drive NK cell apoptosis through the JAK-STAT pathway, upregulating pro-apoptotic genes while suppressing anti-apoptotic factors. JAK inhibitors like tofacitinib, which suppress IFN signaling and restore NK cell counts, may exert therapeutic effects by interrupting this apoptotic cascade. These findings suggest that targeting IFN signaling or protecting NK cells from apoptosis may represent novel strategies for treating anti-MDA5^+^ DM-ILD.

This study has certain limitations. First, the analysis was performed primarily on PBMCs, whereas the primary site of pathology in ILD is the lung. Consequently, the peripheral immune signature may not fully reflect local immunological processes in the pulmonary environment. Second, the bioinformatic analysis and experimental validation were conducted using a limited number of single-cell samples, and the restricted cohort size may reduce statistical power, potentially leading to an oversight of biologically relevant effects. Third, although we demonstrated broad activation of the IFN pathway, the predominant cellular source of IFN production remains unconfirmed. While re-analysis of our single-cell data suggests that monocytes and dendritic cells are likely key contributors, this hypothesis requires further experimental validation. Finally, we only preliminarily established the association between IFN stimulation and increased apoptotic signaling accompanied by reduced NK cell counts in peripheral blood, without performing functional validation of the specific apoptotic pathways involved.

In summary, this study delineates the peripheral immune landscape of anti-MDA5^+^ DM, revealing a distinct signature associated with ILD. Moving beyond established clinical correlations, we provide the first mechanistic evidence that an IFN-rich microenvironment drives NK cell apoptosis, substantiated by single-cell transcriptomics, flow cytometry, and *in vitro* functional validation. These findings introduce a pathogenic model in which dysregulated IFN signaling actively shapes NK cell fate, contributing to peripheral loss and potentially compromising immune regulation. Such insights can inform therapeutic strategies aimed at modulating IFN responses or protecting NK cells from apoptosis to improve clinical outcomes in ILD patients. Subsequent studies can prioritize identifying the cellular sources of pathogenic IFN production and elucidating the precise molecular cascades linking IFN exposure to NK cell death, which will help in the development of novel therapeutic strategies aimed at improving clinical outcomes for patients.

## Data Availability

All data are accessible in NODE (https://www.biosino.org/node) with the accession number OEP00006720 or through the URL: https://www.biosino.org/node/project/detail/OEP00006720.
