# Supplementary material for: Exhaustion of NK cells and interferon activation in anti-MDA5+ dermatomyositis are associated and determine the development of ILD
Source: Front Immunol. 2025 Dec 8;16:1697803. doi: 10.3389/fimmu.2025.1697803 (PMC12719299; doi:10.3389/fimmu.2025.1697803)
Supplement: Supplementary file 1 [file DataSheet1.docx]

**Supplementary Data S1**

*HRCT scoring criteria*

For the sample validation cohort, HRCT results were scored according to the classification method on a scale of 1 to 6. The system classified the dominant radiologic pattern as follows: Grade 1: normal attenuation; Grade 2: ground-glass attenuation without obscuration of bronchial and vascular margins; Grade 3: consolidation without traction bronchiectasis or bronchiolectasis; Grade 4: ground-glass attenuation with traction bronchiectasis or bronchiolectasis; Grade 5: consolidation with traction bronchiectasis or bronchiolectasis; Grade 6: honeycombing. The lungs were divided into upper, middle, and lower zones on both sides, with each zone assessed independently. Abnormality extent was visually estimated as a percentage of affected lung parenchyma, rounded to the nearest 5%. Each zone's score was calculated by multiplying this percentage by the grade (1-6). The average score of the six zones determined each patient's total abnormality score, used to assess ILD severity.

*Single-cell RNA sequencing and data processing*

Raw sequencing data were processed using Cell Ranger (10x Genomics) with the GRCh38-1.2.0 reference genome. Low-quality cells were filtered based on the following thresholds: mitochondrial gene content <10%, detected genes between 200 and 5,000 per cell, and UMI counts ≥500. Potential doublets were identified and removed using DoubletFinder with an expected doublet rate of 10% and 20 principal components. Seurat was employed for data normalization (scaling factor = 10,000), identification of the top 2,000 variable genes, and dimensionality reduction using UMAP with 20 principal components, setting the cell clustering resolution parameter to 0.6.

*Differential expression of genes*

Differential gene expression analysis and visualization of high-throughput data were carried out using R packages such as Seurat, ggplot2, dplyr, and ggrepel. The analysis utilized the FindMarkers function, with a minimum detection fraction of 0.1, a log2 fold change threshold of 0.25, and a P-value threshold of 0.05 to identify significantly differentially expressed genes. Genes with an adjusted P-value of less than 0.05 and an absolute log2 fold change (log2FC) greater than 0.25 were considered significantly upregulated or downregulated.

*Gene set enrichment analysis*

GO and KEGG pathway enrichment analyses were performed using the clusterProfiler package in R, employing the enrichGO and enrichKEGG functions. GO analysis assessed the enrichment of differentially expressed genes (DEGs) across three main categories: Biological Processes (BP), Cellular Components (CC), and Molecular Functions (MF). Gene Set Variation Analysis (GSVA) allows for the assessment of pathway activity changes in an unsupervised manner across the sample population. The method transforms the expression data from a gene-centric to a gene-set-centric perspective, enabling the identification of significant pathway in different samples or groups. Gene Set Enrichment Analysis (GSEA) calculated the Normalized Enrichment Score (NES) based on the Enrichment Score (ES) and subsequently estimated the False Discovery Rate (FDR) and p-values adjusted by the Benjamini-Hochberg (BH) method for each gene set. Gene sets were considered significantly enriched if they met the criteria of FDR < 25%, p-value < 0.05, and |NES| > 1. Reference gene sets were sourced from the MSigDB database (h.all.v2023.2.Hs.symbols.gmt file). Enrichment analyses were conducted using the clusterProfiler, GSVA, GSEABase packages in R, and visualization was facilitated by the enrichplot and GseaVis packages. The UCell algorithm was employed to evaluate the activity of predefined gene sets at the single-cell level. UCell calculates enrichment scores for each individual cell based on the rank of gene expression within its transcriptome. The method uses the Mann-Whitney U statistic to test whether genes in a signature set are enriched toward the top of the ranked list. Resulting scores are normalized between 0 and 1, with higher values indicating stronger signature enrichment. In this study, the irGSEA and UCell packages were used to compute and visualize the enrichment scores for specified gene sets.

*PPI analysis*

Protein-protein interaction (PPI) networks were analyzed using Cytoscape software. The cytoHubba plugin was utilized to identify hub genes among differentially expressed genes using the Maximal Clique Centrality (MCC) algorithm. The top 15 core genes with the highest MCC scores were identified and displayed, offering insights into the network's central components relevant to our research focus.

**Supplementary Table 1 basic characteristics of the validation cohorts**

| **Characteristics** | **Cohort 1  (n=54)** | **Cohort 2  (n=48)** | **Cohort 3  (n=48)** | **P value** |
| --- | --- | --- | --- | --- |
| age, years | 53 (44-60.25) | 48 (42-55) | 52 (42-59) | 0.301 |
| Male, n (%) | 36 (66.7) | 29 (60.7) | 42 (62.7) | 0.446 |
| course_month | 4 (1-9.25) | 6 (1-16) |  | 0.349 |
| ILD, n (%) | 54 (100) | 25 (53.2) |  |  |
| HRCT score | 142.91 (128.33-165.71) |  |  |  |
| **Cytokine, pg/ml** |  |  |  |  |
| IL1-β | 1.36 (0.82-2.3) |  |  |  |
| IL-2 | 1.3 (0.68-1.84) |  |  |  |
| IL-4 | 0.42 (0.74-2.0) |  |  |  |
| IL-5 | 1.07 (0.78-1.32) |  |  |  |
| IL-6 | 5.2 (2.58-12.04) |  |  |  |
| IL-8 | 11.77 (4.95-86.68) |  |  |  |
| IL-10 | 3.0 (2.03-4.38) |  |  |  |
| IL12-P70 | 1.69 (0.92-2.73) |  |  |  |
| IL-17A | 3.68 (1.36-9.96) |  |  |  |
| TNF-α | 1.94 (1.05-2.87) |  |  |  |
| IFN-α | 2.74 (1.86-10.90) |  |  |  |
| INF-γ | 1.69 (0.92-2.47) |  |  |  |

**Except where otherwise indicated, values are shown as the medians (interquartile range).**

Cohort 1: Used for the correlation analysis between HRCT scores and serum cytokine levels.

Cohort 2: Used for the validation of cell apoptosis proportions via flow cytometry.

Cohort 3: Comprising healthy controls, which served as the source of PBMCs for the in vitro plasma stimulation experiments.

**Supplementary Figure**


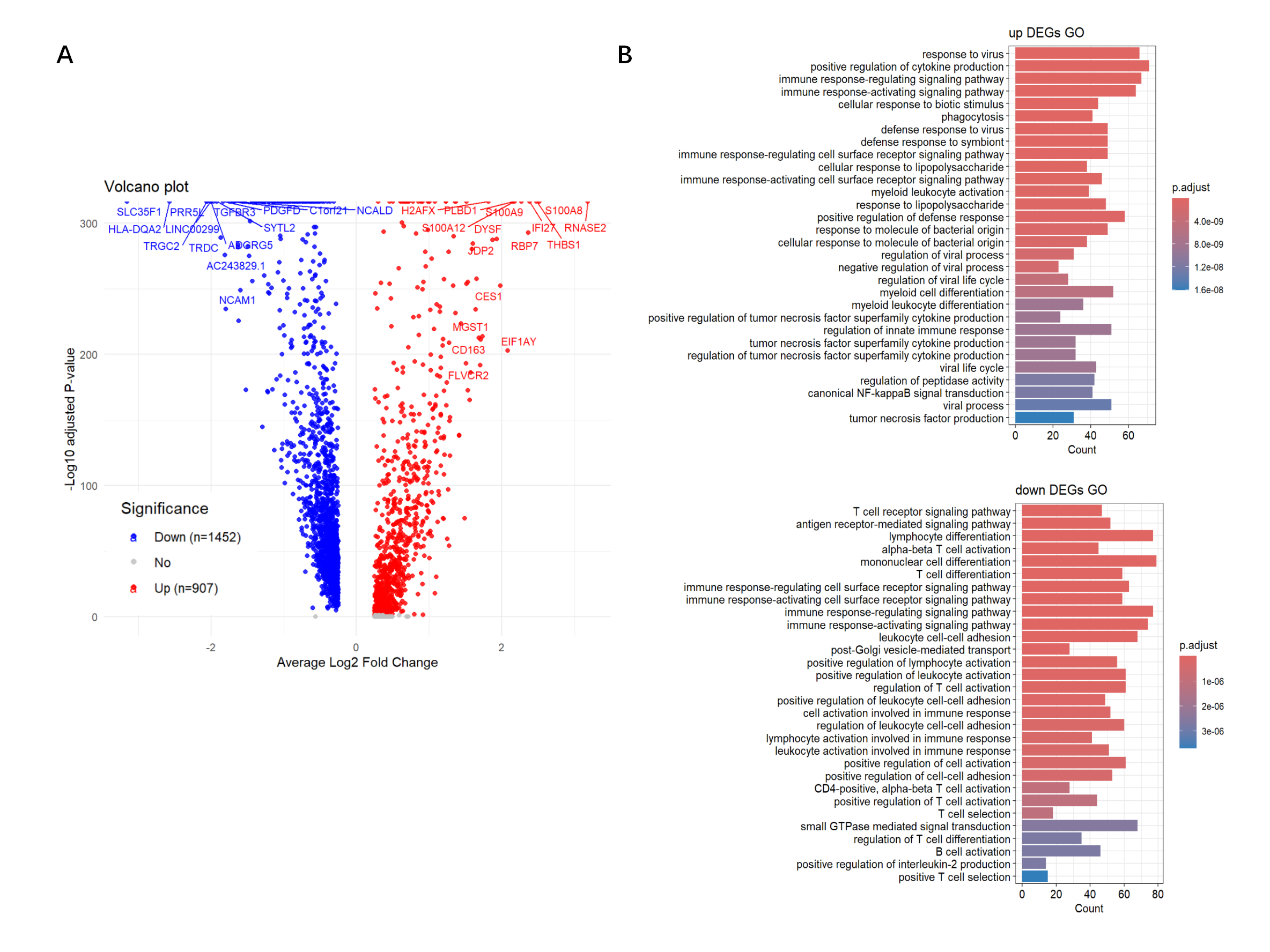


**Supplementary Figure S1** Differential gene expression and functional enrichment in the ILD group.

(A) Volcano plot of differential genes, with upregulated genes in red and downregulated genes in blue. (B) The top panel shows the GO enrichment results for upregulated differential genes, and the bottom panel shows the GO enrichment results for downregulated differential genes.


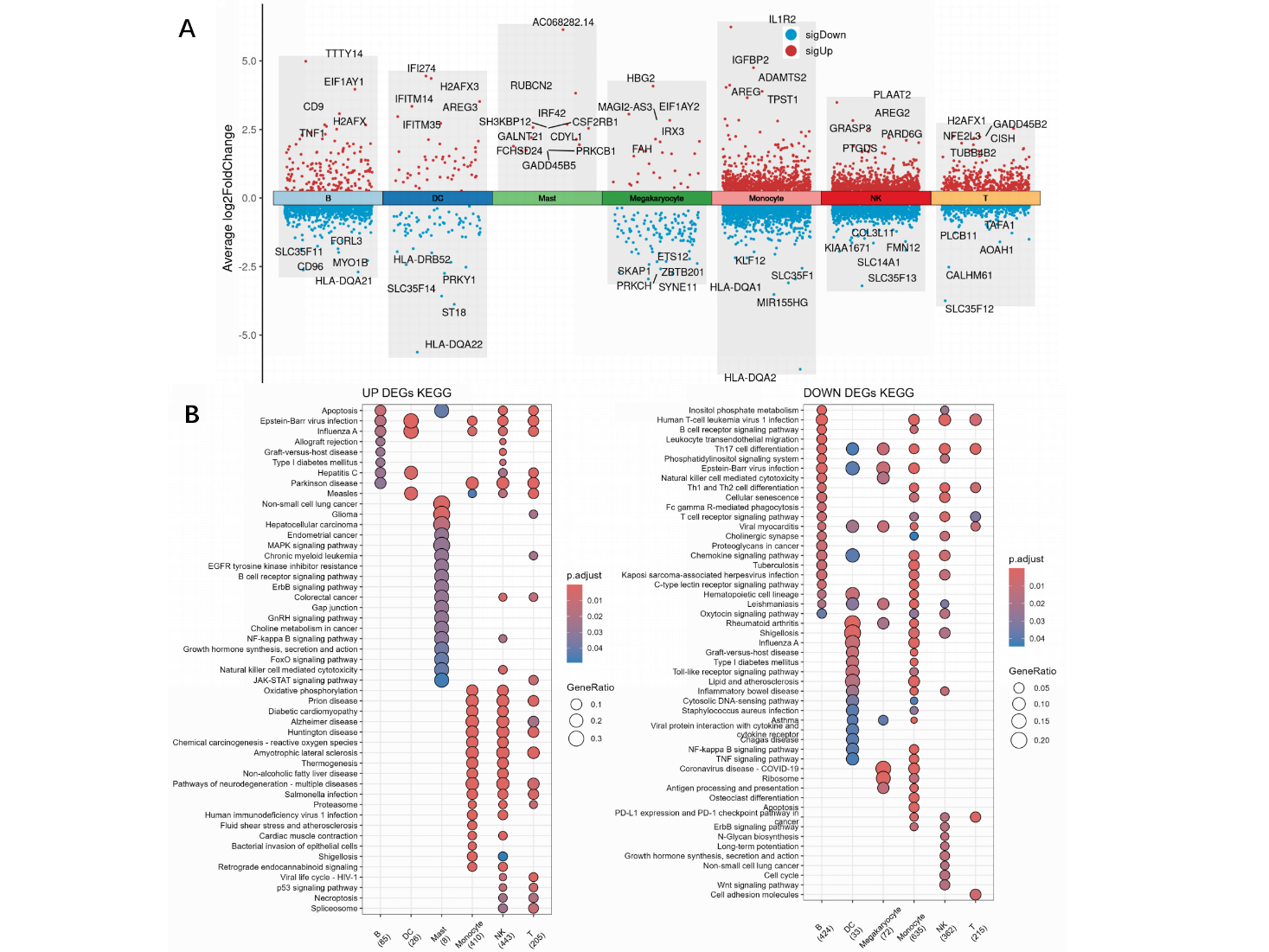


**Supplementary Figure S2** Cell-subpopulation DEG profiling.

(A) Volcano plots showing differentially expressed genes between groups for each cell subset. The top 10 differentially expressed genes are labeled in the plot. (B) Bubble plots of GO enrichment analysis for upregulated and downregulated differentially expressed genes between groups for each cell subset.


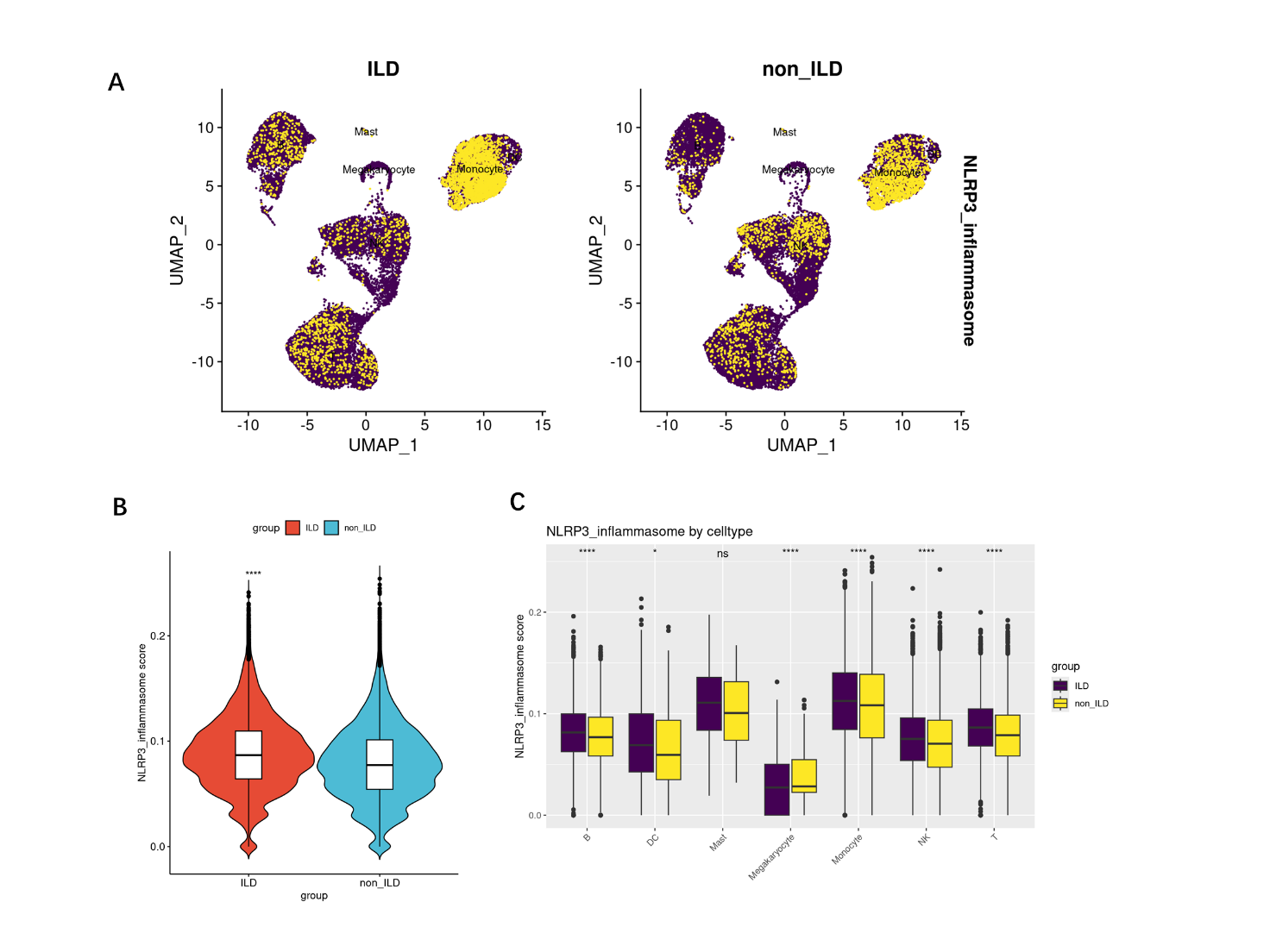
**Supplementary Figure S3** NLRP3 inflammasome Gene Set Scoring. (A) Ucell calculation of enrichment scores for NLRP3 inflammasome pathway. The yellower the color, the higher the density score, indicating a higher enrichment score. (B) Violin plot of comparison of NLRP3 inflammasome gene set scores across groups. (C) Boxplot of differences in NLRP3 inflammasome scores among cell subpopulations.


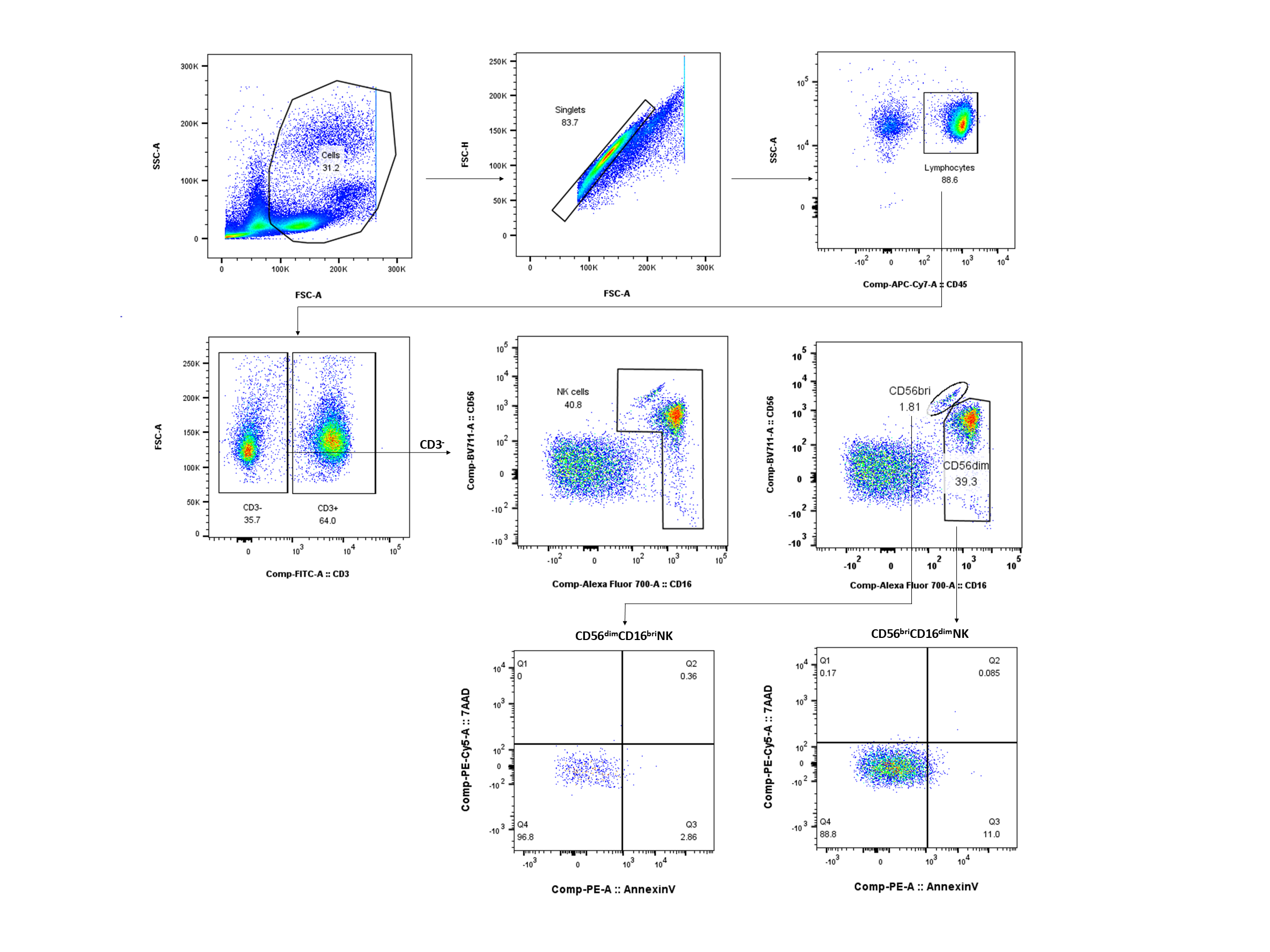


**Supplementary Figure S4** Gating strategy schematic for flow cytometry analysis of PBMC.


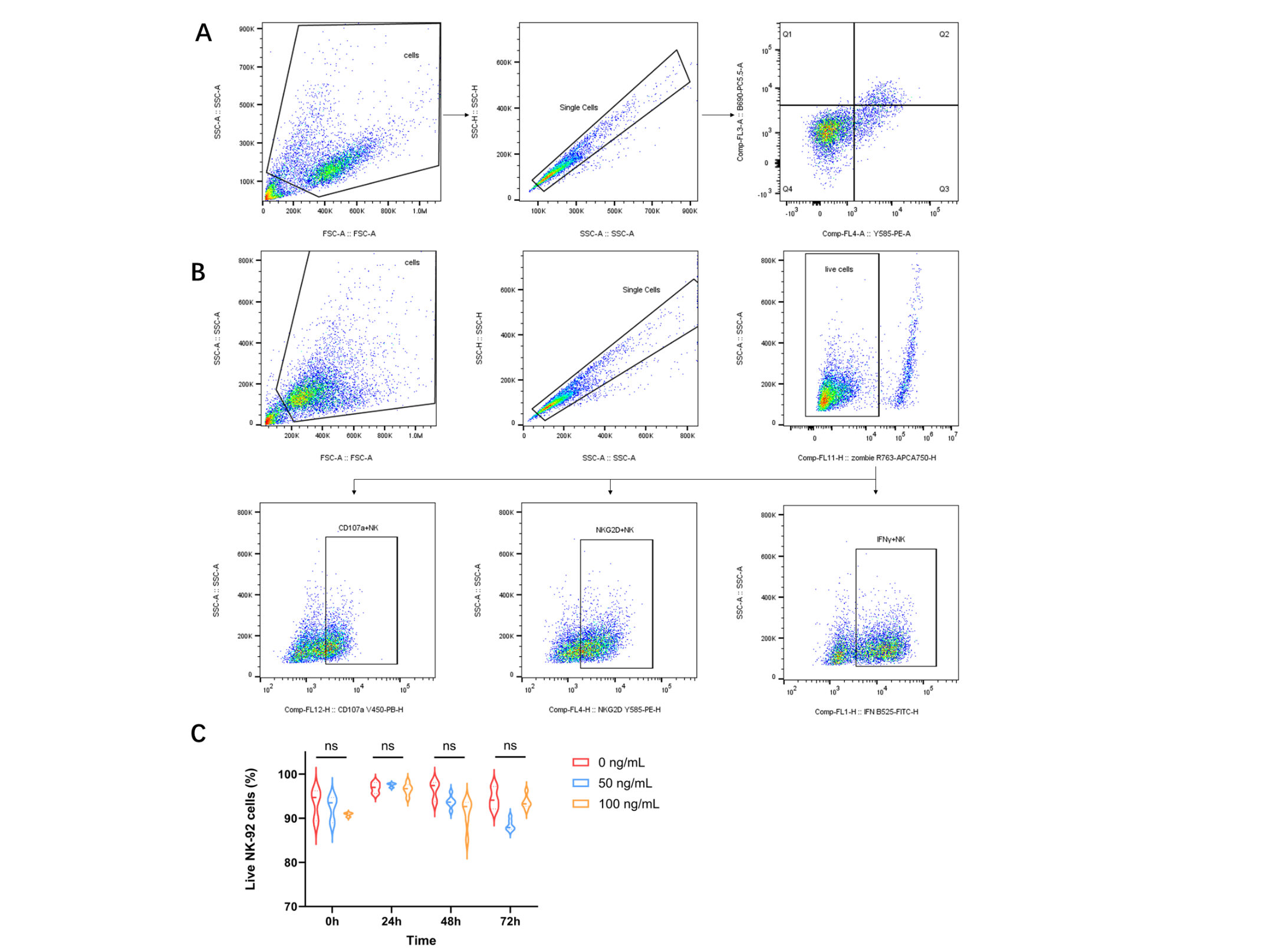
**Supplementary Figure S5** Assessment of NK-92 cell viability under IFN-α stimulation. (A) Gating strategy schematic for flow cytometry analysis of apoptotic NK-92 cells. (B) Gating strategy schematic for flow cytometry analysis of NKG2D^+^/CD107a^+^/IFN-γ^+^ NK-92 cells. (C) Effects of different IFN concentrations and treatment durations on NK-92 cell viability. Data are presented as violin plots showing the median with interquartile range (IQR). Statistical analysis was performed using the Kruskal-Wallis test. No significant differences were observed among the groups.
